# Determinants of Weight Gain among Adult Tuberculosis Patients during Intensive Phase in Debre Markos Town Public Health Facilities, Northwest Ethiopia, 2020: Unmatched Case-Control Study

**DOI:** 10.1155/2022/6325633

**Published:** 2022-03-31

**Authors:** Belsity Temesgen Meselu, Birhanu Barud Demelie, Tigist Adeb Shedie

**Affiliations:** ^1^Midwifery Department, Debre Markos University, Debremarkos, Amhara, Ethiopia; ^2^Amhara Development Association, Debremarkos, Amhara, Ethiopia; ^3^Civics Department, Debre Markos University, Amhara, Ethiopia

## Abstract

**Background:**

Ethiopia is one of the highest tuberculosis burden countries globally, and tuberculosis is one of the most pressing health problems nationally. Weight gain during treatment is the main indicator of good treatment outcome, but there is no adequate information regarding the factors that affect weight gain in Ethiopia.

**Objective:**

The objective of this study was to identify determinants of weight gain among adult tuberculosis patients during the intensive phase, in Debre Markos town public health institutions Northwest Ethiopia, 2020.

**Methods:**

Unmatched case-control study was conducted in Debre Markos town public health facilities with a total sample size of 236. Cases (clients who got weight) and controls (clients who did not get weight) were enrolled in the study consecutively, and data were collected using standardized questionnaires. Data were entered through Epi-Data version 4.2 and exported to SPSS version 25 for analysis. Bivariable analysis was done, and all independent variables that had *p* < 0.25 were entered into multivariable binary logistic regression analysis. Finally, independent variables which were significantly associated with weight gain at *p* < 0.05 were considered determinant factors of weight gain.

**Result:**

Pulmonary tuberculosis (AOR: 5 (95% CI: 2.3, 11.2)), monitoring by health professionals (AOR: 3.7 (1.6, 8.4)), ≥18.5 baseline body mass index (AOR: 3.4 (95% CI: 1.6, 7.3)), parasitic disease (AOR: 3.2 (95% CI: 1.3, 7.99)), <30 days duration of illness before start of treatment (AOR: 2.8 (95% CI: 1.2, 6.1)), and human immune virus/acquired immune deficiency syndrome (AOR: 3.3 (95% CI: 1.2, 9.1)) were independently associated with weight gain compared to their counterpart.

**Conclusion:**

Type of tuberculosis, monitoring by health professionals, baseline status, parasitic disease, duration of illness before start of treatment, and human immune virus/acquired immune deficiency syndrome were with the determinants of weight gain. Therefore, early detection, support and supervision, and attention for comorbidity are mandatory during antituberculosis treatment.

## 1. Introduction

Tuberculosis (TB) is an airborne disease caused by Mycobacterium tuberculosis (MTB) and other closely related Mycobacterium species. Mycobacterium tuberculosis is carried in airborne particles, called droplet nuclei, of 1–5 microns in diameter [1]. Tuberculosis affects individuals of all ages and both sexes globally. However, TB is found to be concentrated among certain high-risk groups due to either higher risk of progression if infected or living in settings where there is an increased risk of transmission. Globally, TB is one of the top 10 causes of death and the leading cause from a single infectious agent more than the human immune deficiency virus (HIV) [2].

Among HIV-negative people, tuberculosis caused an estimated 1.3 million deaths. There were an extra 300,000 deaths from TB among HIV-positive humans. Undernutrition increases the risk of TB that can lead to malnutrition. Weight loss among TB cases can be caused by several factors, including reduced food intake due to loss of appetite [3]. After achieving the global and national targets set for 2015 under the “STOP TB strategy” Ethiopia has articulated an increased political dedication by joining global efforts to end the TB epidemic by 2030 and using “END TB STRATEGY” to decrease the occurrence of TB by 90% and death due to TB by 95% by the end of 2035.

Tuberculosis cases are often malnourished, and malnourished people are at higher risk of developing tuberculosis as their immune system is decreasing. Tuberculosis people often suffer from severe weight loss, which is considered to be immunosuppressive [4]. Tuberclosis patients suffer from weight loss and wasting. Wasting is recognized as a prominent feature of TB and also a major determinant of severity and disease outcome [5]. Malnutrition and tuberculosis are both problems of considerable magnitude in most of the developing areas of the world [6–8]. Malnutrition is the risk factor for developing TB as it leads to an impaired immune system. Cases that have lost weight due to TB are expected to gain weight during successful treatment of the intensive phase [9, 10].

Weight gain is the main indicator of successful treatment outcome; there is scarcity of information or published work about weight gain nationally in this area; rather, studies are on outcome jumping the weight gain during the intensive phase [11, 12].

Tuberculosis is a worldwide health problem of all ages especially in developing countries which is associated with unexplained weight loss. Weight gain is among the mandatory and the first criteria to adjust drug consumption and the main indicator of good treatment outcome in Ethiopia. So, findings from this study will have important policy implications for health intervention program improvement. Moreover, this study will serve as baseline information for those who are interested in further study. The purpose of this study is to identify determinants of weight gain among adult tuberculosis patients during intensive phase in Debre Markos town public health facilities, Northwest Ethiopia, 2020.

## 2. Methods

### 2.1. Study Design, Area, Period, and Population

The case-control study design was conducted in Debre Markos town which is found in East Gojjam Zone, Amhara National Regional State, Ethiopia. Debre Markos is located 300 km far from Addis Ababa with a projected population of 125,634 (BOFED 2020). Health services in the town are provided by one referral hospital, three health centers, seven health posts, seven medium clinics, five specialty clinics, and 14 drug stores and 3 pharmacies. The hospital, all health centers, and three private clinics provide TB treatment programs. The study was conducted between March 05, 2020 and April 30, 2020. Those TB patients who were 18 years of age and above who were registered to start from September 1, 2019 to February 30, 2020 were the study population.

### 2.2. Enrolment of Cases and Controls (Eligibility Criteria)

#### 2.2.1. Case

A case is defined as a person of age group 18 years and above who attended an adult TB treatment clinic in the public health facilities of Debre Markos town from September 1, 2019, to February 30, 2020 with ≥5% weight gain at the end of the intensive phase of treatment.

#### 2.2.2. Control

Control is defined as a person of age group 18 years and above who attended adult TB treatment clinic in the public health facilities of Debre Markos town from September 1, 2019, to February 30, 2020 with <5% weight gain after starting.

#### 2.2.3. Inclusion Criteria

Those patients who were 18 years and above and who were registered from September 1, 2019, to February 30, 2020, were included in this study.

#### 2.2.4. Exclusion Criteria

Those patients who were 18 years and above and who were registered from September 1, 2019, to February 30, 2020, and patients who could not hear were excluded.

#### 2.2.5. Sample Size Determination

The sample size was determined by the Kelsey formula using the Epi-Info version 7.0.8.3 software by considering one variable assumed to bring a difference in the two groups. Sample size calculation was based on the following assumptions: two‐sided confidence level (CI) = 95%, power = 80%, ratio of controls to cases = 1 : 1, and from a similar study conducted in Addis Ababa with working ability as the main predictor of the outcome (weight gain); the percent of controls exposed (proportion of nonweight gain with working ability) was 71% with odds ratio (AOR) of 2.57 (1.42, 4.68) [13]. Thus, the total sample size was 248 by including 10% nonresponse rate (124 cases and 124 controls).

Sample size calculation using Epi-Info software to assess and get different sample sizes from the above one, then the larger sample size used for data collection purpose (Table 1).

#### 2.2.6. Sampling Technique

**Figure 1:**
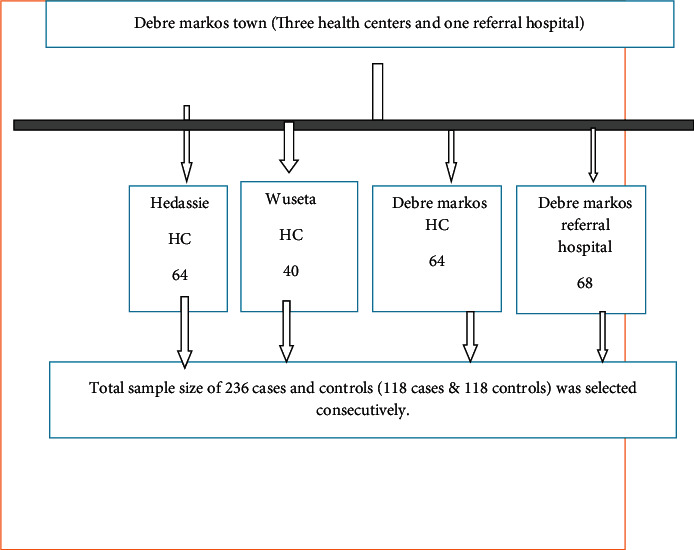
Schematic presentations of sampling process during the intensive phase in Debre Markos town public health facilities, North West Ethiopia, 2020.

Adult TB clinic departments provide services for the age group of fifteen years and above. Both cases and controls were selected from the same adult clinic department visitors (aged 18 years and above) who were diagnosed by health professionals. Weight was taken from the registration book, and cases were selected consecutively from all health facilities during the data collection period until the required sample size was obtained. For each case, the next control was selected consecutively from the same adult TB clinic based on the fulfillment of inclusion and exclusion criteria (Figure 1).

#### 2.2.7. Operational Definitions

A directly observed short-course treatment (DOT) is a service of TB drug distribution under the direct supervision of trained health professionals [2].

Weight gain is increments of weight by ≥5% from the start of treatment after TB treatment started during the intensive phase [8, 14, 15].

Comorbidity diseases are associated with another disease, in addition to tuberculosis in this case.

#### 2.2.8. Data Collection Methods, Quality Management, Processing, and Analysis

Pretest was conducted at Gozamin health center; then, the data were collected using pretested and standardized questionnaires; interviews were conducted by trained data collectors after getting informed consents from the patients. The questionnaire was translated into the local language (Amharic) from its English version then back to English with the guidance of a senior professional. Data collectors were supervised while collecting the data by the principal investigator and other two supervisors, so that technical support was provided accordingly. Data were checked daily for completeness and consistency throughout the data collection period. The strength of association between weight gain and independent variables (covariates) was expressed in odds ratio (OR) with a 95% confidence interval. Bivariable analysis was done, and all variables that were found to have *p* value < 0.25 were entered into the multivariable binary logistic regression model. Independent variables that were significant at *p* value < 0.05 levels in the multiple logistic regression models were declared as determinant factors of weight gain. Finally, the result of the study was presented by narration and tables.

## 3. Result

### 3.1. Sociodemographic Characteristics

Participants enrolled in the study were 248 (124 cases and 124 controls) with a 236 response for both cases and controls. The median age of cases was 30± interquartile range (IQR = 18) and the median age of controls was 27± (IQR = 16). About 55 (46.6%) of cases and 63 (53.4%) controls were male. About 42 (35.6%) of the cases and 31 (26.3%) of the controls were married. From the respondents, 115 (97.5) of cases and 109 (92.4) of controls were Amhara (Table 2).

### 3.2. Clinical Profile of TB Patients

In the clinical related factors, about 62 (52.5%) of cases and 27 (22.9%) of controls were pulmonary positive cases, and 108 (91.5%) cases and 103 (87.3%) cases were new. From the total 236 cases, 88 (74.65%) cases and 33 (28%) controls were starting treatment within a month illness starts. Of which 95 (80.5%) cases and 48 (40.7%) controls were having baseline body mass index ≥ 18.5 kilograms per meter square. From the total study participants, 36 (28 controles and 8 cases) were having HIV/AIDS comorbidity and 49 (39 controls and10 cases) were having previous intestinal parasite history (Table 3).

### 3.3. Supervision and Support-Related Factors

All 236 (100%) study participants were getting their drug from trained health professionals regularly. Of which, 84 (71.2%) of cases and 34 (28.8) controls were getting regular health education from their daily observers. Of those, 93 (78.8%) cases and 35 (29.7%) were getting regular support and supervision (Table 4).

### 3.4. Nutritional Related Factors

From the nutritional related factors, about 89(75.4%) cases and 40(33.4%) controls were getting the meal frequency supplement. From respondents, 31 (45.4%) cases and 46 (63%) controls were getting micronutrient supplementation during treatment (Table 5).

### 3.5. Bivariable and Multivariable Analysis Results

In the bivariable analysis, a total of nine variables (nutritional support, health education, type of TB, baseline status (BMI), support and supervision by health professionals, intestinal parasite disease, duration of illness before start of treatment, and HIV/AIDS) were found to have a *p* value of less than 0.25 and nominated for further analysis in multivariable and Lemeshow goodness-of-fit test at a *p* value greater than 0.05 (used for model fitness test); six variables were showing further association with weight gain at a *p* value less than 0.05. Those are Type of TB, baseline status (BMI), support and supervision by health professionals, intestinal parasite disease, duration of illness before start of treatment, and HIV/AIDS as shown in Table 6.

This study revealed that type of TB was a determinant factor for weight gain and the odds of weight gain for pulmonary TB patients were five times as compared to extrapulmonary TB patients (AOR: 5.0 (95% CI: 2.3, 11.2)).

The odds of weight gain for patients who had ≥18.5 baseline BMI were 3.4 times as compared to patients who had ≤18.49 baseline BMI (AOR: 3.4 (95% CI: 1.6, 7.3)), and the odds of weight gain for patients who starts treatment within 30 days of illness were two point eight times as compared to patients who were starting treatment beyond 30 days (AOR: 2.8 (95% CI: 1.2, 6.1)). The odds of weight gain among patients who got full support from health professionals were 3.7 times as compared to patients who got support partially from health professionals (AOR: 3.7 (95% CI: 1.6, 8.4)). The odds of weight gain for patients who had no parasite disease were 3.2 times as compared to patients who had parasite disease (AOR: 3.2 (95% CI: 1.3, 7.99)). The odds of weight gain for patients who had no HIV/AIDS were 3.3 times as compared to patients who had a history of HIV/AIDS (AOR: 3.3 (95% CI: 1.2, 9.1)) (Table 6).

## 4. Discussion

This study was aimed at identifying the determinant factors of weight gain among adult TB patients during the intensive phase in Debre Markos town public health institutions. Based on this, the study tried to identify the contribution of sociodemographic factors, baseline factors, support and supervision factors, comorbidity related factors, clinical factors, and nutritional factors, and we found six factors independently associated with weight gain including; type of TB, baseline status, support and supervision, disease, duration of illness before start of treatment, and HIV/AIDS.

Type of TB was one of the factors associated with weight gain in this study, in which the odds of weight gain for pulmonary TB were 5 times as compared to extra pulmonary patients, and this finding is in line with studies conducted in the border which indicates weight gain of pulmonary tuberclosis patients was faster than EPTB patient and predictors of clinical response for TB patients were significantly associated with weight gain [16]. A retrospective cohort study conducted in Jimma revealed the odds of weight gain for pulmonary TB patients were more than for extrapulmonary patients [17]. In addition, a study conducted in India showed that the odds of weight gain for pulmonary TB patients were more than extrapulmonary patients [10]. This may be due to the probability of early detection of pulmonary patients than extrapulmonary patients before the immunity deteriorates and also the patient's ability to cope up lowered. But a cross-sectional study conducted in Addis Ababa revealed the type of TB has no association with weight gain [13]. The difference with the result in Addis Ababa may be due to the availability of infrastructures and professionals in Addis that enable the early detection of extrapulmonary patients.

The other independent factor found to be determinant for weight gain was baseline body mass index in this study. The odds of weight gain for patients who had ≥18.5 baselines BMI was 3.4, as compared to patients who have ≤18.49 baseline BMI, and it is in line with studies conducted in Jimma revealed that baseline body mass index values were significantly associated with a change in body weight of patients during treatment [5]. And India revealed that baseline BMI has a significant association with weight gain [10]. This could be due to early detection before emaciation of the patient helps the patient to cope up with the drug and gain weight rapidly.

The other independent determinant factor for weight gain which was identified in this study was support and supervision. The odds of weight gain for fully supervised were 3.7 times as compared to those who supervised partially. This finding is in line with the studies conducted in Mozambique and India which shows that patients who took treatment under supervision were more likely to gain weight [10, 18]. This could be due to the importance of support and supervision to monitor the progress of the patient outcome to take corrective measures.

Duration of illness before the start of treatment was another determinant factor for weight gain during the intensive phase. The odds of weight gain for patients who start treatment within 30 days were two point eight times as compared to patients who were starting treatment beyond 30 days which is in line with a study conducted in Gondar university hospital, in southern Ethiopia, and, in India, all shows that higher risk of unsuccessful outcome was predicted by treatment initiation lately, and lower risk of unsuccessful outcome was predicted by weight gain [9, 19, 20]. This could be due to that early detection before deterioration helps the patient to take the drug safely and cope up with the disease easily.

Comorbidity of HIV/AIDS was the other independent determinant factor for weight gain during treatment. The odds of getting weight for patients who have no HIV/AIDS were 3.3 times as compared to HIV positives. This finding is in line with studies conducted in Jimma and Tanzania revealed HIV status has a significant association with weight gain [15, 18]. This could be due to the effect of HIV/AIDS on immunity as well the effect of pill burden for drug compliance. While a cross-sectional study conducted in Addis Ababa shows TB/HIV coinfection has no significant association with weight gain [13], this could be due to awareness variations and special care given for HIV/AIDS patients in Addis Ababa.

The odds of weight gain for patients who have not any parasitic disease during treatment is 3 times as compared to those who were having parasitic disease, and the finding is in line with studies conducted in Jimma and Tanzania revealed patients who have not any intestinal parasite were getting weight as compared to their counterpart [15, 18]. This could be due to the effect of intestinal parasites on nutrition as well the effect of pill burden for drug compliance.

### 4.1. Limitation of the Study

This study was limited by our inability to cross-check the data provided by the study participants that might lead to information bias and we could not weigh individuals, rather taking the weight from the register.

## 5. Conclusion and Recommendations

The determinants of weight gain among adult TB patients during the intensive phase are type of TB, baseline BMI status, monitoring, intestinal parasites, duration of illness before start of treatment, and HIV/AIDS. Since duration of illness before start of treatment affects weight gain among adult tuberculosis patients, early visiting of health facilities as the symptoms of TB seen is mandatory and also since comorbidities have a great effect on weight gain, tuberculosis screening and getting proper treatment for comorbidities are important for better treatment outcome. A high index of suspicions and diagnosis certainty are mandatory; for cases that need a referral, a timely referral is mandatory. Monitoring of patients during treatment is mandatory and special care is mandatory for patients with comorbidities like monitoring of the drug burden and compliance. For better treatment outcomes, early start of treatment is important. So, highly detectable instruments should be available at all institutions with trained experts. In addition to this, giving training for professionals about considering and treating comorbidities is mandatory.

## Figures and Tables

**Table 1:** Determinant factors of weight gain among adult TB patients during intensive phase taken from previous studies, 2020.

Determinant factors	The proportion among controls and cases	OR	Sample size			Source
			Cases	Controls	Total	
Working ability	*P*1 = 98.3% *P*2 = 71%	2.57	113	113	226	[13]
Counseling	*P*1 = 64.5% *P*2 = 51%	2.2	109	109	218	[13]
Supervision	*P*1 = 83.5% *P*2 = 62.8%	2.57	93	93	186	[9]

^∗^ *P*2: proportion of controls with exposure; *P*1: proportion of cases with exposure A. Then, adding 10% none response rate, the total sample size was 248 (124 cases and 124 controls).

**Table 2:** Sociodemographic factors of study participants during an intensive phase in Debre Markos town public health facilities, Northwest Ethiopia, 2020 (*n* = 118 cases and 118 controls).

Factors	Category	Case	Control	Total
		*n* (%)	*n* (%)	*n* (%)
Sex of patient	Male	55 (46.6)	63 (53.4)	118 (50.0)
	Female	63 (53.4)	55 (46.6)	118 (50.0)
Age of patient (years)	18-40 years	93 (78.8)	88 (74.6)	181 (76.7)
	41-60 years	20 (16.9)	18 (15.3)	38 (16.1)
	≥61 years	5 (4.2)	12 (10.2)	17 (7.2)
Marital status of parents	Married	42 (35.6)	31 (26.3)	73 (30.9)
	Single	43 (36.4)	48 (40.7))	91 (38.6)
	Divorced	31 (26.3)	31 (26.3)	62 (26.3)
	Widowed	2 (1.7)	8 (6.8)	10 (4.2)
Ethnicity	Amhara	115 (97.5)	109 (92.4)	224 (94.9)
	Others	3 (2.4)	9 (7.6)	12 (5.1)
Educational level	<primary education	27 (22.8)	33 (28)	60 (25.4)
	Primary education	39 (33.1)	44 (37.3)	83 (35.2)
	≥secondary education	52 (44.1)	41 (34.7)	93 (39.4)
Occupation	Farmer	1 (0.8)	14(11.9)	15(6.4)
	Housewife	4(3.4)	4(3.4)	8(3.4)
	Civil servant	40(33.9)	35(29.7)	75(31.8)
	Merchant	42(35.6)	42(35.6)	84(35.6)
	Daily laborer	31(26.3)	23(19.5)	54(22.9)
Religion	Orthodox	118 (100)	98 (83.1)	216 (91.5)
	Others	0 (0)	20 (16.9)	20 (8.5)
Residence	Urban	116 (98.3)	80 (67.8)	196 (83.1)
	Rural	2 (1.7)	38 (32.2)	40 (16.9)

**Table 3:** Clinical related factors during an intensive phase in Debre Markos town public health facilities, Northwest Ethiopia, 2020.

Factors	Category	Case	Control	Total
		*n* (%)	*n* (%)	*n* (%)
Type of TB	Smear-positive pulmonary TB	62 (52.5)	27 (22.9)	89 (37.7)
	Smear-negative pulmonary TB	20 (16.9)	35 (29.7)	55 (23.3)
	Extra pulmonary	36 (30.5)	56 (47.5)	92 (39)
Category	New	108 (91.5)	103 (87.3)	211 (89.4)
	Relapse	8 (6.8)	10 (8.5)	18 (7.6)
	Transfer in	2 (1.7)	5 (4.2)	7 (3)
Duration of illness before treatment	Within 30 days	88 (74.6)	33 (28)	121 (51)
	Beyond 30 days	30 (25.4)	85 (72)	115 (48.7)
Baseline status (BMI)	≤18.49	23 (19.5)	70 (59.3)	93 (39.4)
	≥18.5	95 (80.5)	48 (40.7)	143 (60.6)
HIV/AIDS	Yes	8 (6.8)	28 (23.7)	36 (15.3)
	No	110 (93.1)	90 (76.3)	200 (84.7)
History of previous intestinile parasite	Yes	10 (8.5)	39 (33.1)	49 (20.8)
	No	108 (91.5)	79 (66.9)	87 (79.2)

**Table 4:** Supervision and support-related factors of study participants in Debre Markos town public health facilities, Northwest Ethiopia, 2020.

Factors	Category	Case	Control	Total
		*n* (%)	*n* (%)	*n* (%)
Health education on TB treatment	Yes	84 (71.2)	34 (28.8)	118 (50)
	No	34 (28.8)	84 (71.2)	118 (50)
Monitoring by health professionals	Fully partially	93 (78.8)	35 (29.7)	128 (54.2)
		25 (21.2)	83 (70.3)	108 (45.8)
Material support from family members	Fully partially	72 (61)	60 (50.8)	132 (55.9)
		46 (38.9)	58 (49.2)	104 (44.1)
Psychological support from the community	Fully partially	70 (61)	62 (50.8)	132 (55.9)
		48 (38.9)	56 (49.2)	104 (44.1)

**Table 5:** Nutritional related factors of study participants during the intensive phase in Debre Markos town public health facilities, Northwest Ethiopia, 2020.

Factors	Category	Case	Control	Total
		*n* (%)	*n* (%)	*n* (%)
Meal frequency	4 times and above ≤3 time	89 (75.4), 29 (24.6)	40 (33.9), 78 (66.1)	129 (54.7), 107(45.3)
Micronutrient supplement^∗^	Yes	31 (45.6)	46 (63)	77 (32.6)
	No	87 (73.8)	72 (70)	159 (67.4)

^∗^Suplementation of vitamins and minirals.

**Table 6:** Multivariable analysis result of determinants of weight gain among adult TB patients during the intensive phase in Debre Markos town public health facilities, Northwest Ethiopia, 2020.

Baseline status (BMI)	≥18.5	95 (80.5)	48 (40.7)	6.0 (3.4, 10.8)^∗^	3.4 (1.6, 7.3)^∗∗^
	≤18.49	23 (19.5)	70 (59.3)	1	
Duration of illness	Within 30 days	88 (74.6)	33 (28)	7.8 (4.2, 13.4)^∗^	2.8 (1.2, 6.1)^∗∗^
	Beyond 30 days	30 (25.4	85 (72)	1	
Support & supervision	Fully	93 (78.8)	35 (29.7)	8.8 (4.9, 15.9)^∗^	3.7(1.6, 8.4)^∗∗^
	Partially	25 (21.2)	83 (70.3)	1	1
Type of TB	Pulmonary	62 (52.5)	27 (22.9)	3.7 (2.1, 6.5)^∗^	5.0 (2.3, 11.2)^∗∗^
	Extra pulmonary	56 (47.5)	91 (77.1)	1	1
HIV/AIDS	No	110 (93.2)	90 (76.3)	4.3 (1.9, 9.8)^∗^	3.3 (1.2, 9.1)^∗∗^
	Yes	8 (6.8)	28 (23.7)	1	
Parasite	No	108 (91.5)	79 (66.9)	5.3 (2.5, 11.3)^∗^	3.2 (1.3, 7.99)^∗∗^
	Yes	10 (8.5)	39(33.1)	1	
Health education	Yes	84 (71.2)	34 (28.8)	6.1(3.5, 10.7)^∗^	0.8 (0.3, 2.2)
	No	34 (28.8)	84 (71.2)	1	1
Meal frequency	≥4 times	89 (75.4)	40 (33.9)	4 (2.3, 7.3)^∗^	1.2 (0.97, 2.7)
	≤3 times	29 (24.6)	78 (66.1)	1	1

^∗^Nominated for multivariable analysis. ^∗∗^Significant at multivariable analysis at *p* < 0.05.

## Data Availability

The data that support the findings of this study are available to the corresponding authors upon reasonable request.

## Abbreviations

BMI: Body mass index
CI: Confidence interval
COPD: Chronic obstructive pulmonary disease
DOTS: Directly observed therapy short course
DR: Drug-resistant
EPT: Extra pulmonary tuberculosis
HBCS: High-burden countries
HIV: Human immune deficiency virus
MTB: Mycobacterium tuberculosis
NGO: Nongovernmental organizations
OR: Odds ratio
PTB: Pulmonary tuberculosis
RR: Relative risk
TB: Tuberculosis
WHO: World Health Organization.
